# Non-Suicidal Self-Injury among Adolescents: Effect of Knowledge, Attitudes, Role Perceptions, and Barriers in Mental Health Care on Teachers’ Responses

**DOI:** 10.3390/bs14070617

**Published:** 2024-07-20

**Authors:** Inbar Levkovich, Batel Stregolev

**Affiliations:** 1 Faculty of Education, Faculty of Education, Tel-Hai Academic College, Kiryat Shmona 12208, Israel; 2 Faculty of Graduate Studies, Oranim Academic College, Kiryat Tivon 36006, Israel; batel.finer@gmail.com

**Keywords:** non-suicidal, self-injury, teachers, mental health, knowledge, attitudes, self-injury, role perception, barriers

## Abstract

Background: Non-suicidal self-injury (NSSI) involves the deliberate harm of one’s body without the intent to commit suicide and is particularly concerning among adolescents. Teachers play a critical role as gatekeepers in identifying and addressing self-harm, underscoring the importance of their knowledge and response strategies in this area. This study explored how teachers’ knowledge, attitudes towards NSSI, perceived roles, and workplace barriers affect their responses to students exhibiting NSSI behaviors. Methods: A cross-sectional survey was conducted among 203 middle and high school teachers in Israel. Data were collected during July and August 2023 using six validated questionnaires. Results: Higher levels of teacher knowledge, positive attitudes, and strong role perceptions correlated with more effective responses to NSSI, whereas increased workplace barriers tended to diminish response efficacy. Positive correlations emerged between role perception and both knowledge and attitudes, whereas negative correlations emerged between workplace barriers, attitudes, and role perceptions. Teaching experience moderated the impact of role perception and workplace barriers on responses. Significant differences were observed between regular and special education settings, although no differences were noted in referral rates or years of seniority. Conclusions: These findings suggest that enhancing teacher knowledge and attitudes towards NSSI, while addressing workplace barriers, can improve response efficacy in educational settings.

## 1. Introduction

Non-suicidal self-injury (NSSI) is defined as intentionally causing harm to one’s body without suicidal intent. It typically manifests through scratching, cutting, head banging, and burning the skin, which leaves marks or causes tissue damage [1,2]. The most common NSSI behavior includes cutting the skin on the hands and thighs [3,4]. The mainstream view is that NSSI may be a strategy for regulating negative emotions [5]. Non-suicidal self-harm is particularly worrisome among adolescents [6], with an estimated prevalence of 18% in this age group [7]. The lifetime prevalence estimates of NSSI in young adolescents (aged 10–14) have been estimated at around 7–8%, with an increase in prevalence from 14 years onwards [8].

Non-suicidal self-injury (NSSI) Is notably prevalent during adolescence, a particularly vulnerable period for the emergence and progression of mental health issues and risky behaviors [9]. According to the theoretical framework established by Nock and Prinstein [10], NSSI may function as a maladaptive coping mechanism to evade distressed emotional states [11], mitigate negative emotions (e.g., anxiety and depression), and express emotions to others [6]. Both intrapersonal factors (e.g., heightened psychological arousal, internalizing symptoms, and emotion dysregulation) and interpersonal factors (e.g., perceived social support from parents and peers) are deemed essential for the initiation and perpetuation of NSSI behavior over time [12]. These factors constitute vulnerabilities that may hinder adolescents’ ability to manage and cope with challenges or stressful events, thereby increasing the risk of engaging in risky behaviors, such as NSSI, to regulate their emotional experience [10].

Furthermore, NSSI is associated with psychiatric morbidity. Studies have indicated that adolescents suffering from depression, anxiety, borderline personality disorder, and impulsivity are more likely to engage in non-suicidal self-harm [13,14]. The literature has also identified life events that may increase the risk of self-harm among adolescents. Many adolescents who engage in non-suicidal self-harm report past adversity, such as experiences of physical abuse, childhood neglect, and sexual or emotional abuse during childhood [4,6]. Other interpersonal factors include conflicts between family members, difficulties in social relationships, and coping with losses [14].

Non-suicidal self-harm has significant negative consequences for the mental health of those who engage in it [15]. Adolescents who self-harm often experience difficult emotions such as guilt, shame, and an exacerbation of the depression and anxiety that led to the self-harm [11,13]. Additionally, these adolescents frequently face social difficulties, including social stigma related to their mental state and inappropriate reactions from their peers [8]. Consequently, they may feel ostracized and socially isolated [12]. Furthermore, it has been found that adolescents who engage in non-suicidal self-harm are at an increased risk of suicide [16].

Despite the prevalence and inherent risks of NSSI, teenagers often delay seeking professional help. Studies have shown that less than a third of adolescents who engage in non-suicidal self-harm seek assistance [13,17,18]. One contributing factor may be negative attitudes toward non-suicidal self-injury held by peers, family members, and educators in their environment [15,19]. These attitudes can lead to misinterpretations of adolescents’ behaviors and unhelpful reactions from their surroundings [17,20].

Teachers and educational staff sometimes harbor erroneous attitudes toward non-suicidal self-harm. Such attitudes can reinforce students’ perceptions that adults around them do not understand their issues, thereby reducing the likelihood they will seek help. Furthermore, even if students do decide to seek help, the attitudes of their teachers may influence their treatment, the approach and quality of the care provided, and even the outcomes of professional intervention [17].

### 1.1. Teachers’ Perceptions of Non-Suicidal Self-Injury in the Schools

The close relationship between teachers and students enables teachers to be among the first to identify warning signs of non-suicidal self-harm among adolescents. Indeed, teachers are well-positioned to create a safe and supportive classroom environment [16,21]. Yet secondary school teachers have reported a lack of knowledge and training in recognizing and addressing non-suicidal self-injury [22]. Insufficient knowledge in this area may hinder the ability of educational professionals to identify, diagnose, and treat adolescents who engage in non-suicidal self-harm [22,23]. Additionally, this gap in knowledge can lead to misunderstandings and stigmas regarding adolescents and their parents dealing with such issues [24], thereby reinforcing their feelings of loneliness [25]. Teachers have also reported feeling insecure and anxious when faced with adolescents who engage in self-harm, coupled with a sense of helplessness in their ability to provide appropriate responses and assistance [26,27].

Research has indicated that professionals’ attitudes toward adolescents who engage in non-suicidal self-injury significantly influence how they manage this behavior [28]. Studies have identified a spectrum of attitudes towards self-harm among those close to affected teenagers. Some professionals exhibit responsibility, empathy, and support, thus facilitating meaningful and open relationships that positively affect the quality of care [29,30]. Conversely, self-injurious behavior is often perceived negatively, either as a mental illness or as attention-seeking behavior, leading to despair and criticism among observers who are unaware of the underlying issues [15,31]. Such misperceptions foster stigma, contribute to the adolescents’ sense of shame, isolation, and exacerbated distress, and potentially intensify their self-harming behavior [12,32,33]. Such attitudes can also undermine the effectiveness of treatment and support for those who engage in NSSI.

Many educators have acknowledged the importance of their role in addressing students’ mental health needs [34,35]. They recognize their responsibility to monitor the well-being of their pupils, identify those who are vulnerable, and facilitate referrals for necessary support and assistance. Despite this recognition, teachers report ambiguity regarding their expected contributions and express a lack of requisite training and skills in mental health support [36]. Furthermore, they mention a need for adequate professional support from mental health professionals, which is contingent on the availability of sufficient time and resources within the educational system [34,37]. These findings highlight the critical gap between the expectations placed on teachers and the support they are given, underscoring the need for clearer guidelines and better resource allocation to empower teachers to fulfill their role in student mental health care effectively.

Understanding the complexity of teachers’ roles in identifying and responding to students experiencing mental distress requires an examination of inherent barriers [34,36]. A primary challenge is the difficulty in detecting issues such as self-harm among adolescents, who often conceal their struggles from the educational staff [1,38]. Additionally, addressing mental health in educational settings can contribute to significant emotional and functional stress among teachers, compounding their daily challenges [39]. Teachers have also reported deficiencies in their training in intervention strategies and the lack of a structured plan for prevention, hampering their ability to support students and engage with their parents effectively [17,37,40]. Further complicating these issues are systemic barriers such as the limited availability of specialized staff, insufficient funding for mental health services, and poor coordination with community mental health resources [34]. Studies have also noted that a lack of knowledge, inadequate emotional support for teachers, and reduced resources, including fewer opportunities for consulting with school counselors, are significant obstacles to managing self-harm and other mental health issues among adolescents [26,41].

### 1.2. The Current Research

To provide empathetic and effective support for adolescents, mental health and educational professionals must understand the essence, causes, and consequences of non-suicidal self-injury (NSSI) [15,42]. Research has indicated that empathetic responses that validate adolescents’ feelings and coping strategies can mitigate feelings of shame and encourage openness regarding their struggles [43]. By fostering a safe and calm environment, educational counselors can avoid exacerbating the situation through pressure or anger, thus facilitating more constructive interaction and promoting cooperative behavior among adolescents. This approach also emphasizes the importance of involving other professionals and guiding adolescents towards further professional help [27,43,44].

The initial response that adolescents receive when disclosing self-harm significantly affects their subsequent handling of the issue. Many adolescents report negative initial encounters, including judgmental reactions and long waits for assistance, which contribute to feeling undervalued [11,17]. Additionally, teachers described their initial reactions as marked by shock and anxiety, complicating their ability to respond effectively [26]. Teachers also pointed to a general lack of clear guidelines and intervention methods for dealing with self-harm at school, leading to feelings of insecurity and helplessness [13,27,37]. Further studies confirm that educators often harbor negative attitudes towards self-harm, characterized by disgust and rejection, adversely affecting the quality of their responses [24,45].

The aim of the present study was to assess: (1) the relationship between teachers’ knowledge, attitudes, barriers, and perceptions of their role in responding to non-suicidal self-harm; and (2) the moderating role of experience working with adolescents who have engaged in non-suicidal self-injury between study variables.

**Hypotheses** **1.** *Levels of knowledge about non-suicidal self-harm, attitudes about non-suicidal self-harm, and perceptions of the teacher’s role in these events will exhibit a positive relationship to the ways in which teachers respond to non-suicidal self-harm.*

**Hypotheses** **2.** *A negative relationship will be found between the level of barriers encountered by teachers working with teenagers who engage in non-suicidal self-injury and educators’ responses to non-suicidal self-injury.*

**Hypotheses** **3.** *Knowledge, attitudes, role perception, and barriers will predict how educators respond to non-suicidal self-injury.*

**Hypotheses** **4.** *Experience working with adolescents who have engaged in non-suicidal self-injury will moderate how knowledge, attitudes, role perceptions, and barriers affect educators’ responses to non-suicidal self-injury.*

## 2. Methods

### 2.1. Research Process

Before the study began, ethical approval was obtained from the university ethics committee (approval number 972023). The data collection instrument was an online questionnaire in Israel. The questionnaires were administered through the Qualtrics online platform (www.qualtrics.com) and distributed via a link shared within dedicated teacher groups on various social media platforms, specifically Facebook, in Hebrew, between 18 July 2023 and 10 August 2023. No incentives were offered to participants. Participants were given an application letter detailing the content of the informed consent form and were required to confirm their consent before accessing the questionnaire. To ensure confidentiality, the questionnaire was designed to be anonymous, omitting identifying information about the participants or their associated schools.

### 2.2. Participants

This study employed a quantitative cross-sectional design, involving 203 secondary school teachers as participants (Table 1). Participants were recruited using non-probability consecutive sampling, whereby all individuals meeting the inclusion criteria were invited to participate. The inclusion criteria were teachers currently working in either middle or high school settings across Israel, encompassing both the regular and special education sectors. The exclusion criteria ruled out elementary school teachers, substitute teachers, and teachers currently on probation (internships). The participants’ demographic profile revealed an average age of 39.99 years, with a range from 24 to 64 years and a standard deviation of 9.18. The majority of participants were female (77.6%) and married (77.6%). The teachers were distributed across the regular education system (124 teachers, 61.1%) and the special education system (79 teachers, 38.9%). On average, the participating teachers had 12.73 years of teaching experience (range 1 to 35 years) and 8.64 years of experience in their current positions (range 0 to 33 years). A significant proportion of participants (57.6%) reported having experience with students who had engaged in non-suicidal self-harm.

### 2.3. Research Tool

The following validity and reliability measures were used in the study:

Dependent Variable:

*Response Methods: A questionnaire regarding students who engaged in non-suicidal self-injury was administered to teachers* [46]. This instrument has been used in other studies to examine teachers’ responses to non-suicidal self-injury [13,17,29]. The questionnaire included 14 items rated on a 4-point Likert scale ranging from 1 (“do not agree at all”) to 4 (“strongly agree”). A sample item is: “Referring students who engage in self-harm for assessment and treatment at community mental health services is an effective course of action for dealing with them”. Scores were calculated as the average response, with higher scores indicating more effective response methods. In the current study, the internal reliability as measured by Cronbach’s alpha was α = 0.81.

Independent Variables:

*1. Self-Injury Knowledge Questionnaire* [47]: The questionnaire includes ten statements rated on a 5-point Likert scale ranging from 1 (“do not agree at all”) to 5 (“completely agree”). Sample items include: “Self-harm is a release of anger”, “Self-harm expresses emotional pain”, and “Self-harm provides an escape from depression”. The overall score is the average of the responses, with higher scores indicating greater knowledge of non-suicidal self-harm. In the current study, the internal reliability of this questionnaire was α = 0.87.

*2. Attitudes towards non-suicidal self-injury questionnaire* [48]: This questionnaire contains 17 statements, rated on a 5-point Likert scale. A sample item is: “Students who harm themselves usually make me feel angry”. Scores reflect the average response, with higher scores indicating a more positive attitude. In the current study, internal reliability was α = 0.81.

*3. Role Perception in the Field of Mental Health of Students* [49]: This questionnaire consists of 13 statements rated on a 5-point Likert scale. A sample item is: “I believe I can play a role in ensuring that students’ mental health needs are addressed in a timely manner”. The average score indicates the respondent’s perceived role, with higher scores reflecting stronger perceptions. In the current study, the internal reliability was α = 0.82.

*4. Barriers to Work in the Field of Mental Health of Students* [49]: This questionnaire includes 12 statements rated on a scale from 1 to 5. Sample items include: “Lack of adequate training to address the mental health needs of children”, “Lack of coordinated services between schools and the community”, and “Lack of funding for school-based mental health services”. Higher average scores indicate greater perceived barriers. The questionnaire’s internal reliability in the current study was α = 0.84.

*5. Demographic and Personal Details:* This questionnaire collected information on participants’ gender, age, marital status, education level, years of seniority in the education system, type of work setting, and experience working with teenagers who have engaged in self-harm.

### 2.4. Statistical Analysis

The sample size was calculated using G*Power (version 3.1). With a high power of 0.95, an alpha level of 0.05, and a low effect size of f^2^ = 0.02, the calculation indicated the need for a larger sample. However, by adjusting the effect size to a moderate level of f^2^ = 0.15, a regression analysis with six predictors requires 146 participants. The data were analyzed using SPSS version 27 (IBM, Armonk, NY, USA). Descriptive statistics were utilized to detail the demographic characteristics of the participants and the research variables. Pearson correlations were computed to evaluate the associations among the research variables. The strength of the correlations was categorized as follows: 0–0.20 indicating a weak correlation, 0.21–0.50 indicating a moderate correlation, 0.51–0.80 indicating a good correlation, and 0.81–1.00 indicating an excellent correlation. Furthermore, a stepwise linear regression analysis was used to assess the predictive power of the variables by incorporating the control variables as needed. To explore the moderation hypothesis, four moderating models were implemented using Hayes’ [50] PROCESS version 3.5 macro, with bootstrap confidence based on 5000 random samples. The analysis was conducted at a 95% confidence level. This rigorous analytical approach ensured a robust examination of the proposed hypotheses within the research framework. Before conducting the statistical analyses, the study variables were examined for outliers, which were defined as values exceeding 4 standard deviations from the mean (z scores > |4|) [51].

## 3. Results

The mean values of the research variables—knowledge, attitudes, role perceptions, barriers, and response methods—were significantly higher than the scale midpoint (Table 2). This finding suggests that the participants had a generally positive orientation toward managing non-suicidal self-injuries.

In support of the first hypothesis, a significant positive correlation was observed between response methods and the following variables: knowledge of non-suicidal self-harm (r = 0.46, *p* < 0.001), attitudes towards non-suicidal self-harm (r = 0.37, *p* < 0.001), and role perceptions (r = 0.66, *p* < 0.001). These findings corroborate the hypothesis that higher knowledge levels, positive attitudes, and clear role perceptions are associated with more effective response methods.

Furthermore, in support of the second hypothesis, a significant negative correlation was found between barriers and response methods (r = −0.29, *p* < 0.001). This finding indicates that higher perceived barriers are associated with less effective response methods, partially validating the associated hypotheses (Table 2).

A stepwise linear regression analysis was conducted to assess the third research hypothesis, according to which demographic and research variables will predict how participants respond to non-suicidal self-injury. The dependent variable in the analysis was the nature of the response to non-suicidal self-injuries. The model comprised two steps: The first step included demographic variables such as seniority and school setting, and the second step added research variables such as knowledge, attitudes, role perception, and barriers.

The results of the first step yielded a significant model [F(2, 200) = 10.43, *p* < 0.001], explaining 9% of the variance in the response methods. The inclusion of the research variables in the second step significantly enhanced the model’s explanatory power [F(6, 196) = 40.09, *p* < 0.001], accounting for 55% of the variance in the response methods. A detailed examination of the second step revealed that knowledge (*t* = 5.61, *p* <0.001), attitudes (*t* = 3.03, *p* < 0.005), and role perception (*t* = 8.98, *p* <0.001) were significant predictors. High levels of knowledge, positive attitudes, and strong role perceptions were associated with more effective response methods (Table 3).

Four moderating models were implemented to evaluate whether experience working with adolescents who engage in non-suicidal self-harm moderates the relationship between various independent variables and response strategies. These models utilized linear regression analyses with bootstrapping procedures as outlined by the PROCESS method [50]. Specifically, Model 1 of Hayes’ [50] PROCESS macro was employed. This analysis used bootstrapping with 5000 samples, and was conducted at a 95% confidence level, as detailed in Hayes’ PROCESS version 3.5. According to this method, if the confidence interval does not include 0, the interaction is considered significant. The independent variables considered were knowledge, positive attitudes, perceptions, and barriers, and the dependent variable was response method.

In the first model, which focused on knowledge as the independent variable, the overall model proved to be significant [F(3, 199) = 25.34, *p* < 0.001]. However, the interaction term between knowledge and experience working with adolescents did not reach statistical significance (*p* = n.s.), indicating that experience had no moderating effect on the relationship between knowledge and response method.

The second model examined the influence of positive attitudes. While the overall model was significant [F(3, 199) = 22.26, *p* < 0.001], the interaction between positive attitudes and experience did not achieve significance (*p* = n.s.), suggesting that experience does not moderate the impact of positive attitudes on the response method.

The third model explored the moderating effect of experience working with adolescents who engaged in non-suicidal self-harm on the relationship between role perception and ways of responding. The overall model was statistically significant [F(3, 199) = 75.02, *p* < 0.001], demonstrating a robust relationship between these variables (Table 4).

The predictive power of role perception and response strategies, with experience as the moderating factor, was weakened but still significant and positive (B = 0.22, SE = 0.07, *p* < 0.005; LLCI = 0.08, ULCI = 0.36, *p* < 0.001).

**Figure 1:**
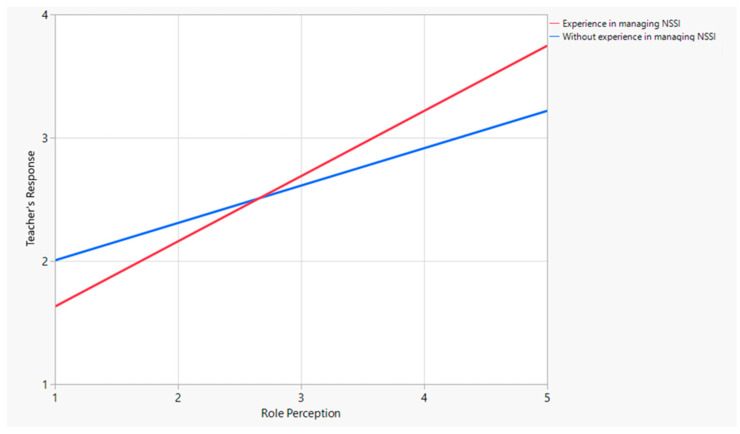
Interaction between teachers with experience managing non-suicidal self-harm and role perception.

The interaction term between role perception and experience working with affected adolescents was found to be statistically significant (*t* = 3.16, *p* < 0.005). This result indicates that among teachers who had experience managing non-suicidal self-harm, role perception had a stronger influence on their response methods than among teachers without such experience. As illustrated in Figure 1, among teachers with experience managing non-suicidal self-harm, role perception had a significantly stronger influence on their response methods compared to teachers without such experience. This interaction is depicted by the steeper slope in the line, which represents teachers with experience. This finding underscores the importance of experience in enhancing the impact of role perceptions on response strategies among educators.

**Figure 2:**
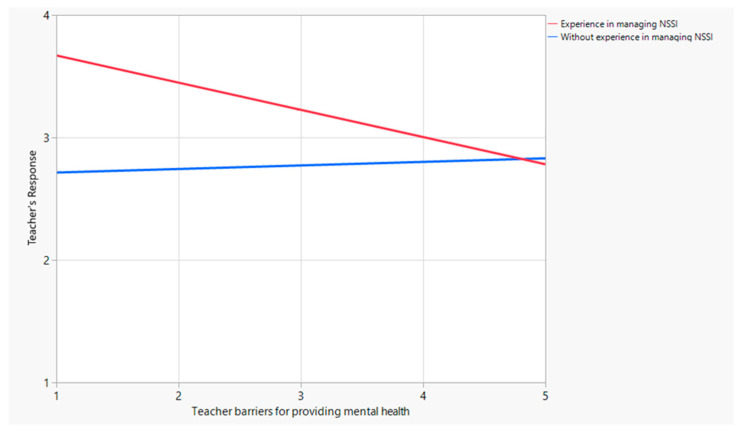
Interaction between teachers with experience managing non-suicidal self-harm and teachers’ barriers for providing mental health.

**Figure 3:**
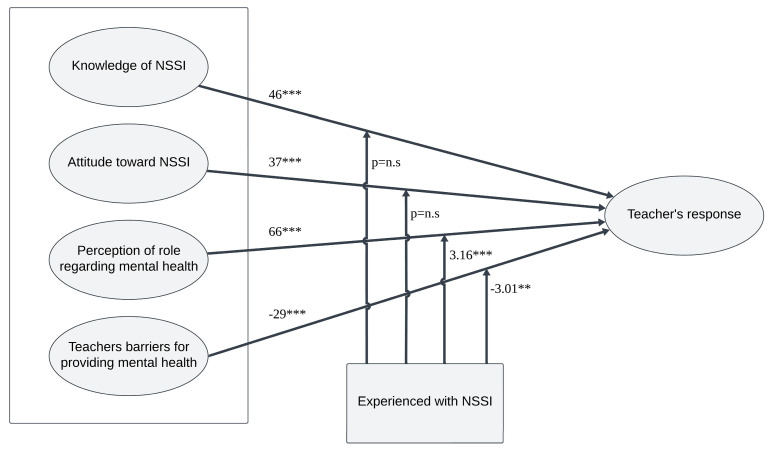
The research model. ** *p* < 0.005, *** *p* < 0.001, n.s: not significant.

The fourth model investigated the moderating effects of experience working with adolescents who engaged in non-suicidal self-harm on the relationship between perceived barriers and response strategies. The results indicated that the overall model was statistically significant [F(3, 199) = 21.75, *p* < 0.001], suggesting a strong association between these factors. One crucial finding is that the interaction term between perceived barriers and experience was statistically significant (*t* = −3.01, *p* < 0.005). This finding reveals that among teachers with experience dealing with non-suicidal self-harm, the presence of numerous barriers had a significant impact on a limited range of response methods. Conversely, among teachers without this experience, perceived barriers exhibited no significant effect on response methods. Thus, this hypothesis is partially supported (Table 5). The predictive power of barriers and response strategies, with experience as the moderating factor, was weakened but remained significant and negative (B = −0.25, SE = 0.08, *p* < 0.005; LLCI = −0.41, ULCI = −0.08, *p* < 0.001). As illustrated in Figure 2, for teachers with experience in managing non-suicidal self-harm, the presence of numerous barriers significantly impacted the range of response methods, thereby limiting their effectiveness. Figure 3 Presenting the Research Model and Findings.

## 4. Discussion

The objective of this study was to investigate how teachers’ knowledge and attitudes towards non-suicidal self-harm (NSSI), their perceptions of their role, and the barriers they face affect their response strategies in managing student mental health. Additionally, experience working with adolescents who engaged in NSSI was explored as a moderating factor in the relationship among knowledge, attitudes, role perception, barriers, and response methods.

The findings of this study revealed a positive correlation between knowledge of NSSI and the effectiveness of response strategies. This correlation aligns with prior research indicating that inadequate responses to NSSI among adolescents are more prevalent among teachers who report insufficient knowledge and understanding of the phenomenon [26,36]. Social Cognitive Theory posits that confidence in one’s ability, or sense of self-efficacy, is a crucial determinant of behavior [52]. When applied to NSSI, both positive attitudes and belief in one’s capability to address NSSI in a school setting are important determinants of whether school staff can effectively serve as gatekeepers [44]. Insufficient knowledge and a lack of appropriate skills can lead to stress and confusion within educational teams, which may compromise their ability to identify NSSI behaviors and respond appropriately to affected students. Furthermore, this deficiency can hinder the educational team’s ability to manage these cases effectively, potentially leading to the avoidance of treating these adolescents [22,23,27].

In the current study, a positive correlation was observed between attitudes towards non-suicidal self-injury (NSSI) and response methods to NSSI. In a qualitative study conducted in Israel among 27 teachers, it was found that, although the teachers felt insecure and lacked experience, skills, and knowledge to help students who engage in self-injurious behaviors, their responses were characterized by empathy, concern, and compassion towards the adolescents [27]. Empathic, compassionate, and supportive attitudes among professionals have been shown to significantly enhance the quality of care provided to adolescents who are coping with NSSI [30]. Such attitudes also increase the likelihood that adolescents will seek and accept help [29]. Conversely, negative, judgmental, and critical attitudes negatively impact the quality of care and support available to these adolescents, potentially exacerbating their distress and worsening their self-injurious behavior [28,31,33]. This relationship can be elucidated through the lens of the theory of planned behavior [53], which posits that attitudes influence behaviors via a process of deliberate decision-making. Behavior is the result of behavioral intention, implying that to predict the likelihood of a specific behavior, one must understand the attitude toward that behavior. Self-injury can significantly predict psychological distress and the propensity for suicidal behavior. Empathy demonstrated by teachers towards adolescents has the potential to alleviate psychological suffering and facilitate the formation of a deep and enduring bond [13,16].

The findings of this study also revealed a positive relationship between teachers’ perceptions of their role in managing students’ mental health and their responses to non-suicidal self-harm (NSSI). The results suggest that teachers who have positive perceptions of their mental health roles demonstrate empathy towards their students and utilize effective response strategies, supporting prior research [34]. Conversely, teachers who perceive their role negatively tend to avoid addressing students’ mental health issues [28].

The concept of “emotional availability” may explain this finding. This concept highlights the significance of positive role perception in the context of student mental health. Emotional availability, which was originally used as a measure for evaluating the quality of emotional communication between parents and children, describes a supportive environment that facilitates autonomy and emotional support, akin to a nurturing maternal presence [54,55]. Similarly, if teachers perceive that their role is to be highly available and integral in addressing students’ issues, including self-harm, they are more likely to engage in supportive and effective responses.

Furthermore, the study found that barriers within the educational setting negatively affected teachers’ responses to NSSI. The participating teachers reported deficiencies in knowledge and training and the absence of tailored response plans, all of which had a detrimental impact on their ability to manage students’ mental health issues effectively [34,40,41]. A study conducted in Israel examined educators who worked with adolescents who had attempted suicide. The results indicated that these educators felt hurt, vulnerable, and insecure addressing the needs of these adolescents. They also experienced a heightened sense of responsibility [56]. Additionally, emotional strain, ongoing stress in dealing with students with mental health challenges, and structural obstacles such as limited budgets and insufficient staff further complicate this issue [1,3,17,38]. These findings underscore the critical need for improved resources and support systems as an effective means of enhancing teachers’ capabilities in responding to students’ mental health needs.

The research model accounted for 55% of the variance in predicting methods of responding to non-suicidal self-harm (NSSI), with significant contributions from three variables: knowledge, attitudes, and teachers’ role perceptions. Teachers who are well-informed about NSSI and have been trained to manage such situations possess confidence in their ability to identify, respond to, and support affected adolescents effectively and demonstrate enhanced response capabilities [1,17]. Furthermore, the literature suggests that teachers’ attitudes significantly influence how adolescents are treated. Educators and counselors with negative attitudes towards adolescents who engage in NSSI often report feelings of shock, rejection, and a tendency to avoid involvement with them [11,57].

This study also revealed that working with adolescents who have engaged in NSSI moderates the relationship between role perception, encountered barriers, and response methods. Teachers with prior experience coping with NSSI were found to be more adept at responding effectively to these situations, underscoring the value of practical experience in enhancing quality of care [20,57]. Kolb’s [57] Experiential Learning Theory posits that learning is a process whereby knowledge is created through the transformation of experience. Teachers with prior experience in dealing with NSSI have gone through the stages of concrete experience, reflective observation, abstract conceptualization, and active experimentation. These stages allow teachers to learn from their past interactions with students engaging in NSSI, refining their skills and strategies over time. This cyclical process of learning enables them to respond more effectively in future situations involving NSSI [13]. This finding emphasizes the need for targeted training and experiential learning opportunities for educators to better manage and support students in dealing with mental health challenges [27].

### 4.1. Study Limitations

This study has several notable limitations. First, it employs a cross-sectional design, in which data collection occurs at a single point in time. This design limits the ability to track changes over time or establish causality. Further studies involving the long-term monitoring of participants are necessary. Second, the study utilized non-probability sampling, which restricts the generalizability of the findings to broader populations. Third, the online distribution of the questionnaire, specifically via social networks, means that only those with access to these networks were able to participate, potentially biasing the sample. Fourth, the predominance of female participants in the study raises questions about gender differences in responses. The inclusion of more male participants might have yielded different insights regarding responses to adolescents engaging in non-suicidal self-injury. Moreover, the study examined the discrepancies between attitudes and actual behavior. It is recommended that future studies incorporate additional research tools to gain a more comprehensive understanding. In addition, our findings may be specific to teachers in Israel and may not be generalizable to other populations. Finally, several factors were not examined in this study, including the effects of gender, culture, and personal or family members’ experiences with non-suicidal self-harm. These aspects warrant further investigation in future research. 

### 4.2. Practical Recommendations

Based on the findings of this study, several practical recommendations can be made to enhance how teachers respond to adolescents who engage in non-suicidal self-harm. There is a clear need for developing and implementing comprehensive knowledge and training programs on self-harm that are specifically tailored for educators. In addition, intervention programs facilitated by psychologists and educational consultants should be established. Workshops conducted by educational consultants could be beneficial in strengthening teachers’ role perceptions and increasing their awareness of mental health issues.

It is also crucial to address systemic barriers within the educational system that affect handling such phenomena; for example, by assessing the resources and support provided to educational institutions for managing self-harm. The proper allocation of budgets for mental health initiatives should be evaluated to ensure that sufficient funding is available. Furthermore, schools should make sure to employ adequately trained emotional support staff, such as school psychologists, educational counselors, and emotional therapists, who are knowledgeable about the proper response to non-suicidal self-injury. These professionals can offer both professional and emotional support to teachers, thus helping them manage such cases effectively.

The potential impact of these results on educational policy and practice is significant. Policymakers should prioritize mental health education and support within schools, integrating comprehensive training programs into the professional development of educators. Schools should establish clear guidelines and protocols for identifying and responding to NSSI, ensuring that all staff are aware of the appropriate steps to take when confronted with such cases. Additionally, fostering a collaborative environment where teachers, mental health professionals, and administrators work together can enhance the overall effectiveness of interventions and support systems.

Specific suggestions include implementing ongoing training programs with regularly scheduled sessions for teachers on identifying and managing NSSI, incorporating the latest research and best practices. Schools should develop clear protocols, establishing and disseminating step-by-step guidelines for teachers to follow when they encounter students engaging in NSSI. Adequate resources should be allocated to ensure that schools have the funding and support needed for mental health initiatives, including hiring additional mental health professionals. Creating support networks for teachers, where they can share experiences, seek advice, and receive emotional support from colleagues and mental health professionals, is also crucial. Finally, enhancing collaboration by promoting a collaborative approach to student mental health, involving regular meetings and communication between teachers, school counselors, psychologists, and parents, can significantly improve the support system for students. By implementing these recommendations, educational institutions can create a more supportive and effective environment for addressing non-suicidal self-injury among adolescents.

## 5. Conclusions

This study aimed to investigate the impact of teachers’ knowledge, attitudes, role perceptions, and workplace barriers on their responses to students engaging in non-suicidal self-injury (NSSI) behaviors. The researchers conducted a cross-sectional survey among 203 middle and high school teachers. The results showed that higher levels of teacher knowledge, positive attitudes, and strong role perceptions were associated with more effective responses to NSSI, whereas increased workplace barriers tended to diminish response efficacy. This study highlights the importance of enhancing teacher training and support programs to improve interventions for students exhibiting NSSI, ultimately enhancing both educational and mental health outcomes.

## Figures and Tables

**Table 1:** Study participants (N = 203).

		f	%
Gender	Male	15	7.4
	Female	188	92.6
Family status	Single	24	13.1
	Married	142	77.6
	Divorced	13	7.1
	Widowed	2	1.1
	Other	2	1.1
Education	BA	73	36.0
	MA	128	63.0
	PHD	2	1.0
Education type	Regular education	124	61.1
	Special education	79	38.9
Past experience with NSSI?	No	86	42.4
	Yes	117	57.6
	Mean	SD	Range
Age	39.99	9.18	24–64
Education seniority	12.73	9.16	1–35

**Table 2:** Means, SDs, and intercorrelations for the study variables (N = 203).

	Mean	SD	Range	1	2	3	4	5
1. Knowledge	3.68	0.78	1–5	-				
2. Attitudes	3.75	0.60	1–5	0.12	-			
3. Perception of Role	3.74	0.61	1–5	0.28 ***	0.32 ***	-		
4. Barriers	3.48	0.71	1–5	−0.12	−0.20 **	−0.31 ***	-	
5. Teacher’s Response	2.99	0.43	1–5	0.46 ***	0.37 ***	0.66 ***	−0.29 ***	-

** *p* < 0.005, *** *p* < 0.001.

**Table 3:** Hierarchical regression for predicting teacher’s response (N = 203).

	B	SE	β	*t*	R^2^	F Model
Step 1						
Education type	0.00	0.00	0.12	1.78	0.09	10.43 ***
Seniority	0.27	0.06	0.30	4.44 ***		
Step 2						
Education type	0.00	0.00	0.00	0.03	0.55	40.09 ***
Seniority	0.02	0.04	0.02	0.47		
Knowledge	0.15	0.02	0.28	5.61 ***		
Attitude	0.11	0.03	0.16	3.03 **		
Perception of Role	0.35	0.04	0.50	8.98 ***		
Barriers	−0.04	0.03	−0.06	−1.29		

** *p* < 0.005, *** *p* < 0.001.

**Table 4:** Findings of regression between role perception and response strategies when experience is the moderating factor (N = 203).

	Coeff	SE	*t*	LLCI	ULCI	R^2^	F Model
Role Perception	0.30	0.05	5.64 ***	0.19	0.40	0.53	75.02 ***
Experienced with NSSI	−0.60	0.26	−2.24 *	−1.13	−0.07		
Perception of Role * Experienced with NSSI	0.22	0.07	3.16 **	0.08	0.36		

* *p* < 0.05 ** *p* < 0.005, *** *p* < 0.001.

**Table 5:** Findings of the regression between barriers and ways of responding when experience is the moderating factor (N = 203).

	Coeff	SE	*t*	LLCI	ULCI	R^2^	F Model
Barriers	0.02	0.06	0.41	0.10	0.16	0.24	21.75 ***
Experienced with NSSI	1.20	0.30	4.02 ***	0.61	1.79		
Barriers * Experienced with NSSI	−0.25	0.08	−3.01 **	−0.41	−0.08		

** *p* < 0.005, *** *p* < 0.001.

## Data Availability

The data used in the study can be made available upon requests addressed to the corresponding author.
